# Three‐dimensional echo planar spectroscopic imaging for differentiation of true progression from pseudoprogression in patients with glioblastoma

**DOI:** 10.1002/nbm.4042

**Published:** 2018-12-17

**Authors:** Gaurav Verma, Sanjeev Chawla, Suyash Mohan, Sumei Wang, MacLean Nasrallah, Sulaiman Sheriff, Arati Desai, Steven Brem, Donald M. O'Rourke, Ronald L. Wolf, Andrew A. Maudsley, Harish Poptani

**Affiliations:** ^1^ Department of Radiology Perelman School of Medicine at the University of Pennsylvania Philadelphia PA USA; ^2^ Department of Pathology and Lab Medicine Perelman School of Medicine at the University of Pennsylvania Philadelphia PA USA; ^3^ Department of Radiology University of Miami Miami FL USA; ^4^ Department of Hematology‐Oncology Perelman School of Medicine at the University of Pennsylvania Philadelphia PA USA; ^5^ Department of Neurosurgery Perelman School of Medicine at the University of Pennsylvania Philadelphia PA USA; ^6^ Department of Cellular and Molecular Physiology University of Liverpool Liverpool UK

**Keywords:** echo planar spectroscopic imaging, glioblastoma, proton MRS, pseudoprogression, true progression, tumor treatment response

## Abstract

Accurate differentiation of true progression (TP) from pseudoprogression (PsP) in patients with glioblastomas (GBMs) is essential for planning adequate treatment and for estimating clinical outcome measures and future prognosis. The purpose of this study was to investigate the utility of three‐dimensional echo planar spectroscopic imaging (3D‐EPSI) in distinguishing TP from PsP in GBM patients. For this institutional review board approved and HIPAA compliant retrospective study, 27 patients with GBM demonstrating enhancing lesions within six months of completion of concurrent chemo‐radiation therapy were included. Of these, 18 were subsequently classified as TP and 9 as PsP based on histological features or follow‐up MRI studies. Parametric maps of choline/creatine (Cho/Cr) and choline/N‐acetylaspartate (Cho/NAA) were computed and co‐registered with post‐contrast *T*_1_‐weighted and FLAIR images. All lesions were segmented into contrast enhancing (CER), immediate peritumoral (IPR), and distal peritumoral (DPR) regions. For each region, Cho/Cr and Cho/NAA ratios were normalized to corresponding metabolite ratios from contralateral normal parenchyma and compared between TP and PsP groups. Logistic regression analyses were performed to obtain the best model to distinguish TP from PsP. Significantly higher Cho/NAA was observed from CER (2.69 ± 1.00 versus 1.56 ± 0.51, *p* = 0.003), IPR (2.31 ± 0.92 versus 1.53 ± 0.56, *p* = 0.030), and DPR (1.80 ± 0.68 versus 1.19 ± 0.28, *p* = 0.035) regions in TP patients compared with those with PsP. Additionally, significantly elevated Cho/Cr (1.74 ± 0.44 versus 1.34 ± 0.26, *p* = 0.023) from CER was observed in TP compared with PsP. When these parameters were incorporated in multivariate regression analyses, a discriminatory model with a sensitivity of 94% and a specificity of 87% was observed in distinguishing TP from PsP. These results indicate the utility of 3D‐EPSI in differentiating TP from PsP with high sensitivity and specificity.

**Abbreviations used**
^1^H MRS: proton MRS
3D: three dimensional
AUC: area under the ROC curve
BW: bandwidth
CCRT: concurrent chemo‐radiation therapy
CER: contrast enhancing region
Cho: choline
Cr: creatine
DPR: distal peritumoral region
EPSI: echo planar spectroscopic imaging
FLAIR: fluid‐attenuated inversion recovery
GBM: glioblastoma
IPR: immediate peritumoral region
MIDAS: Metabolite Imaging and Data Analysis System
MPRAGE: magnetization‐prepared rapid acquisition of gradient echo
NAA: N‐acetylaspartate
*N*_EX_: number of excitations
PsP: pseudoprogression
RANO: Response Assessment in Neuro‐Oncology
rCBV: relative cerebral blood volume
RN: radiation necrosis
ROC: receiver operating characteristic
ROI: region of interest
*T*_E_: echo time
*T*_I_: inversion time
TMZ: temozolomide
TP: true progression
*T*_R_: repetition time
TTFields: tumor treating fields

## 1 INTRODUCTION

Glioblastoma (GBM) is the most common malignant brain tumor in adults, with a dismal prognosis even after aggressive multimodal therapy. The current standard of care for newly diagnosed GBM comprises maximal safe tumor resection followed by radiation therapy with concurrent temozolomide (TMZ) chemotherapy followed by six cycles of adjuvant TMZ.^1^ However, in a subset of cases, contrast enhancement and surrounding *T*_2_/FLAIR (fluid‐attenuated inversion recovery) signal abnormality within the radiation treatment field, at the site of the original tumor or resection margins, is observed within six months of completion of concurrent chemo‐radiation therapy (CCRT). While these imaging findings may represent true progression (TP) of the neoplasm, they may also reflect “treatment effect” or pseudoprogression (PsP) that is mediated by TMZ‐induced increased vascular permeability and inflammatory response.^2^ The incidence of PsP ranges from 28% to 66% in GBM patients undergoing CCRT.^3^ Interestingly, PsP patients are more responsive to TMZ treatment and are associated with a better clinical outcome than TP patients. As a result, PsP patients are closely monitored with short interval follow‐up MRI scans and symptomatically managed with continuation of adjuvant TMZ, whereas TP patients often require invasive and/or alternate therapies.^2^ Therefore, accurate differentiation between TP and PsP lesions is critical for making informed decisions on therapeutic intervention and for prognostication. However, RANO (Response Assessment in Neuro‐Oncology) criterion^4^ based conventional imaging findings are often equivocal in distinguishing between TP and PsP and present a considerable diagnostic challenge.^5^

Several studies have reported the utility of proton MRS (^1^H MRS) for studying brain tumor metabolism^6^, ^7^, ^8^, ^9^, ^10^, ^11^ and in evaluating treatment response in GBM patients treated with anti‐angiogenic agents.^12^ Multiple studies^13^, ^14^, ^15^ have reported the utility of single voxel or single slice multivoxel ^1^H MRS in distinguishing recurrent tumors from radiation necrosis, which may be similar to PsP in histological appearance^16^ but is different in time of onset and degree of severity,^17^ suggesting that ^1^H MRS may also be a useful tool in identifying PsP. A previous study,^18^ using single voxel ^1^H MRS, reported higher choline (Cho) and lower lipid levels in TP than in PsP patients. However, single voxel or single slice multivoxel ^1^H MRS results suffer from incomplete sampling of the neoplasm. Furthermore, both TP and PsP tissues encompass a wide spectrum of histologic features, including areas of tumor as well as treatment effect. The heterogeneous nature of such lesions renders sampling a critically important factor in determining the reliability of ^1^H MRS for accurate identification of TP and PsP. 3D‐echo planar spectroscopic imaging (EPSI) allows acquisition of volumetric metabolite maps with high spatial resolution, minimizing partial volume effects.^19^, ^20^ The potential of 3D‐EPSI has been reported in characterizing glioma grades,^21^ mapping glycine distribution in gliomas,^22^ planning radiation therapy for GBM patients,^23^ identifying residual tumor following radiation therapy,^24^ evaluating response to epigenetic modifying agents in recurrent GBM,^25^ evaluating treatment response to tumor treating fields (TTFields),^26^ and assessing the effect of whole brain radiation therapy on normal brain parenchyma in patients with metastases.^27^ However, its utility in differentiating between TP and PsP has not been investigated.

We hypothesize that sub‐regions of neoplasms reveal different metabolite patterns between TP and PsP. Therefore, the purpose of the current study was to utilize 3D‐EPSI in differentiating TP from PsP in GBM patients following maximal safe resection who demonstrated an enhancing lesion within six months of completion of standard CCRT. A subset of patients also underwent a second 3D‐EPSI approximately 4 weeks after the baseline study to assess evolution of metabolic differences between TP and PsP over time.

## 2 MATERIALS AND METHODS

### 2.1 Patients

This retrospective study was approved by the institutional review board and was compliant with the Health Insurance Portability and Accountability Act, and informed consent was obtained from each patient. The primary inclusion criteria were (a) histologically confirmed diagnosis of GBM, (b) maximal safe surgical resection followed by standard of care CCRT and adjuvant TMZ, (c) new enhancing lesion (>1 cm) in the radiation field within six months of the completion of CCRT, and (d) follow‐up surgery/biopsy or more than 2 follow‐up routine diagnostic MRI scans for a pathological or clinico‐radiological confirmation of TP or PsP. Based upon the inclusion criteria, 27 patients (mean ± standard deviation, 64.2 ± 9.84 years of age; males/females, 14/13) were included. Of these, 18 were TP, including 4 patients who were clinically diagnosed as TP at the time of initial presentation confirmed by an increase in the size of the enhancing lesion at two or more consecutive follow‐up routine MRI studies according to the updated RANO criteria and 14 patients were confirmed to have malignant features on histopathological and immunohistochemical analyses of the re‐resected surgical specimens. In each of these 14 cases with TP, malignant features (increased mitotic activity, endothelial cell proliferation, and presence of pseudopalisading necrosis) accounted for more than 25% of the tumor specimens. Moreover, the presence of nuclear overexpression of p53 protein in the tumor specimens confirmed the presence of TP status; however, absence of positive reactivity for p53 staining did not reject the result. A threshold value of 25% for malignant features was used to classify a tumor specimen as either TP or PsP, keeping curative intent in mind. If the malignant features are more than 25%, the usual clinical course is to subject the patients to repeat surgery and/or alternate therapy at our institution.

The other 9/27 patients were determined to have PsP, defined as stable or decreasing size of the enhancing lesion at two or more consecutive follow‐up diagnostic MRIs (*n* = 7), or having a predominant treatment effect (<25% malignant features) characterized by necrosis, gliosis, fibrosis, vascular hyalinization, microphage infiltration, and dystrophic calcification from the re‐resected tumor specimens (*n* = 2). The clinico‐radiological diagnosis for each patient as TP or PsP on initial presentation was established by a consensus opinion at weekly multidisciplinary neuro‐oncology conference.

After the baseline study, 7/27 patients with TP were subsequently enrolled in various clinical trials and 9/27 patients were lost to follow‐up. The remaining 11/27 patients underwent a repeat 3D‐EPSI study approximately 4 weeks following the baseline study. Data from 3 of these patients were excluded due to poor quality, either due to severe motion artefacts (*n* = 1) or if the raw data were corrupted during the transfer process by the presence of a numeric data type representing an undefined value commonly referred to as “not a number” (NaN, *n* = 2). None of the baseline data suffered from these issues. Of the 8 useable follow‐up MRS data sets, 3 were in TP and 5 in PsP groups.

### 2.2 Data acquisition

All MRI data were acquired on a 3 T Tim Trio MR scanner (Siemens, Erlangen, Germany) equipped with a 12‐channel phased array head coil. The anatomical imaging protocol included a three‐plane scout localizer, axial three‐dimensional (3D)‐*T*_1_‐MPRAGE (magnetization‐prepared rapid acquisition of gradient echo) imaging (*T*_R_/*T*_E_/*T*_I_ = 1620/3.9/950 ms); matrix size = 192 × 256; section thickness = 1 mm; number of sections per slab = 192; flip angle = 15°; number of excitations (*N*_EX_) = 1; bandwidth (BW) = 150 Hz/pixel, an axial *T*_2_‐FLAIR image (*T*_R_/*T*_E_/*T*_I_ = 9420/141/2500 ms); section thickness = 3 mm; number of sections = 60; flip angle 170°; *N*_EX_ = 1; BW = 287 Hz/pixel. *T*_1_‐weighted images were repeated after administration of a standard dose (0.1 mmol/kg) of gadobenate dimeglumine contrast agent (MultiHance; Bracco Diagnostics, Monroe Township, NJ, USA). 3D‐EPSI data were acquired from the entire brain, encompassing both supratentorial and infratentorial brain regions, except for some portions of the frontal brain and brain stem. The total number of voxels covering the brain was in the range of 16200 – 18398. These data were acquired before contrast injection with a spin‐echo sequence^28^ with parallel imaging using generalized autocalibrating partially parallel acquisition and an acceleration factor of 1.6. Acquisition parameters for 3D‐EPSI sequence included *T*_R_/*T*_E_ = 1550/17.6 ms, *N*_EX_ = 1, field of view = 280 × 280 × 180 mm^3^, spatial points = 50 × 50 × 18, voxel size = 5.6 × 5.6 × 10 mm^3^ (0.31 cm^3^), complex points = 512, BW = 625 Hz, and excitation angle = 73°. To reduce lipid signal contamination from the skull and scalp, an inversion recovery prepared lipid nulling pulse sequence with an inversion time of 198 ms was used in addition to employing an outer volume saturation band (50 mm thick) covering the skull base. Acquisition time was about 16 min, including an interleaved water reference acquisition scan obtained using a gradient‐echo acquisition with parameters identical to those for the metabolite imaging except for 20° excitation angle and a 6.3 ms *T*_E_. The water‐unsuppressed scan was used to perform eddy current correction.

### 2.3 Image processing

EPSI data were post‐processed offline using MIDAS (Metabolite Imaging and Data Analysis System) software developed by Maudsley et al.^20^ The raw data were corrected for *B*_0_ field inhomogeneity, and eddy current induced distortions. Additional processing steps included re‐gridding, spatial, and spectral Fourier transformation. The acquired data, with 50 × 50 × 18 resolution, were zero‐filled to a final spatial resolution of 64 × 64 × 32, resulting in a nominal voxel size of 4.3 × 4.3 × 5.6 mm^3^. This was followed by generation of brain masks that defined the brain region and were used to limit the voxels selected for spectral analysis. Additionally, lipid masks generated from subcutaneous lipid signals were used for lipid *k*‐space extrapolation to reduce ringing artifacts. The MIDAS tool allowed evaluation of data quality by generating quality maps. In each case, quality assurance was evaluated by considering the following metrics: Cramer‐Rao lower bounds, line shapes, CSF partial volume contribution, and degree of residual water and lipid signals. The outlier voxels with poor spectral fitting were excluded from further analysis. Finally, parametric maps of Cho/creatine (Cr) and Cho/N‐acetylaspartate (NAA) ratios were computed.

**Figure 1:**
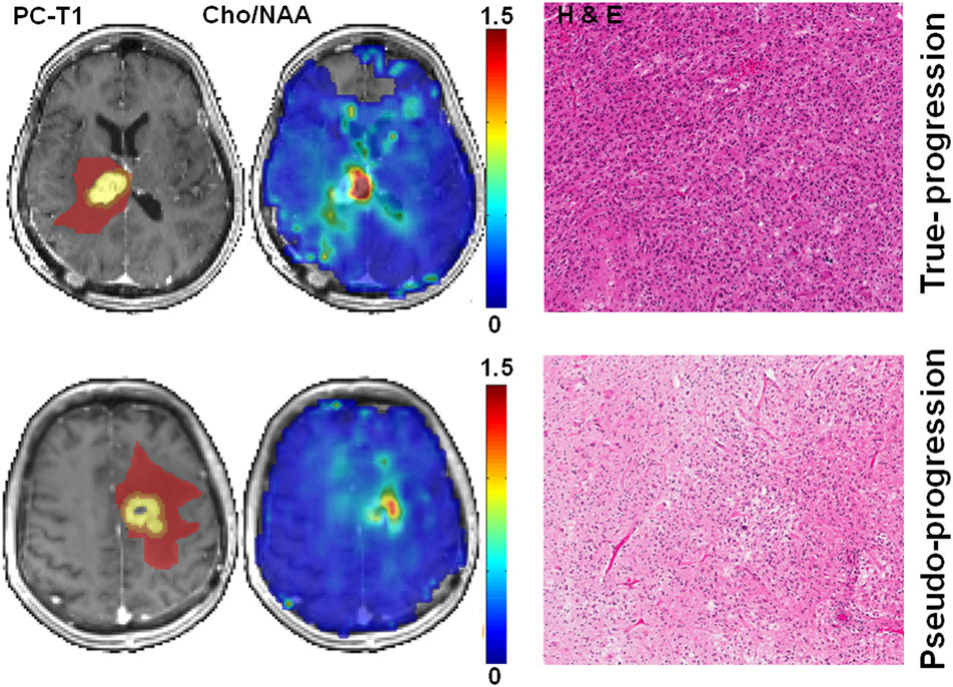
Top row, axial post‐contrast *T*_1_‐weighted image from a patient with TP, demonstrating a neoplasm in the right thalamus infiltrating into the lateral ventricles. The regions of interest (ROIs) are overlaid on the image, with the colors indicating the following defined regions: Yellow, CER; orange, IPR; brown, DPR. The corresponding Cho/NAA map shows areas of elevated Cho/NAA (color bar indicating the distribution of non‐normalized Cho/NAA). A photomicrograph of a histologic section (hematoxylin–eosin stain) shows areas of high tumor cellularity, pseudopalisading necrosis, endothelial proliferation, and increased mitotic activity. Bottom row, axial post‐contrast *T*_1_‐weighted image from a patient with PsP, demonstrating a neoplasm in the left frontal lobe. The different ROIs as defined above are overlaid on the image. The corresponding Cho/NAA map shows reduced Cho/NAA compared with that of the TP case shown above. A photomicrograph of hematoxylin–eosin stain reveals most of the tissue with treatment‐related changes, including extensive geographic necrosis and vascular fibrinoid necrosis

Using custom IDL (Exelis, Boulder, CO, USA) and MATLAB (MathWorks, Natick, MA, USA) based scripts, metabolite ratio maps (Cho/Cr and Cho/NAA) were spatially aligned with post‐contrast *T*_1_‐weighted MPRAGE and FLAIR images. Co‐registration between EPSI maps and imaging was performed using down‐sampled anatomical scan resolutions to yield a 1:1 ratio matching of the voxel size. As shown in Figure 1, each lesion was segmented into contrast enhancing (CER), immediate peritumoral (IPR), and distal peritumoral regions (DPR) using a semi‐automated segmentation algorithm as described previously.^29^ In brief, the CER was defined as the region with enhancement higher than mean + 3SD of the signal intensity from the contralateral white matter. The IPR was arbitrarily chosen as one MRS voxel dimension around the CER. The remaining region of FLAIR abnormality outside of IPR was defined as DPR.

Median Cho/Cr and Cho/NAA values were computed for all voxels within each segmented region and for normal tissue on the contralateral side. The normal brain region was drawn by selecting a volume comparable to the tumor size and at the same slice level while avoiding any voxels affected by lipid contamination or poor signal quality. There were no significant differences (*p* > 0.05) in the Cho/Cr (0.24 ± 0.06 versus 0.28 ± 0.08) and Cho/NAA (0.20 ± 0.07 versus 0.22 ± 0.03) ratios from contralateral normal brain parenchyma between TP and PsP patients. Normalized Cho/Cr and Cho/NAA values were computed by dividing the metabolite ratios from each segmented region of the tumor by the corresponding metabolite ratios from contralateral normal parenchyma to minimize inter‐subject variability and to facilitate unbiased group comparison. All metabolite ratios in tumor regions will henceforth reflect the normalized values throughout the text.

### 2.4 Statistical analysis

Kolmogorov–Smirnov tests were used to determine the nature of data distribution. As the data showed departure from Gaussian distribution, non‐parametric Mann–Whitney *U* tests were performed to assess differences in Cho/Cr and Cho/NAA ratios from different regions of neoplasms between TP and PsP groups. A probability (*p*) value of less than 0.05 was considered significant. Receiver operating characteristic (ROC) curve analyses were performed to evaluate the ability of the metabolite ratios (Cho/Cr and Cho/NAA from CER, IPR, and DPR regions) to discriminate TP from PsP. Optimal threshold values that maximize Youden's index were determined, and the sensitivity, specificity, and area under the ROC curve (AUC) for each parameter were computed. All the metabolite ratios were incorporated into multivariate logistic regression analyses to ascertain the best model for classification. Leave‐one‐out cross‐validation was applied to estimate the accuracy of the model.

For the follow‐up study, Mann–Whitney *U* tests were performed to evaluate the differences in metabolite ratios (Cho/NAA and Cho/Cr) from segmented tumor regions between TP and PsP at the follow‐up time point. Additionally, percent changes ((follow‐up − baseline)/baseline × 100) in the metabolite ratios from the segmented regions of the neoplasms were estimated and were compared between the two groups. All statistical analyses were performed using PASW Statistics, Version 18.0 (IBM, Armonk, NY, USA).

## 3 RESULTS

In general, good quality EPSI data were obtained from all patients at baseline. Quality map analysis revealed that more than 60% of the voxels were of good quality from all patients. The majority of voxels with insufficient quality suffered from a broad spectral linewidth due to field distortions caused by poor *B*_0_ homogeneity and magnetic susceptibility artifacts. The poor‐quality spectra were mainly found in the frontal lobe and in the brain stem regions.

**Figure 2:**
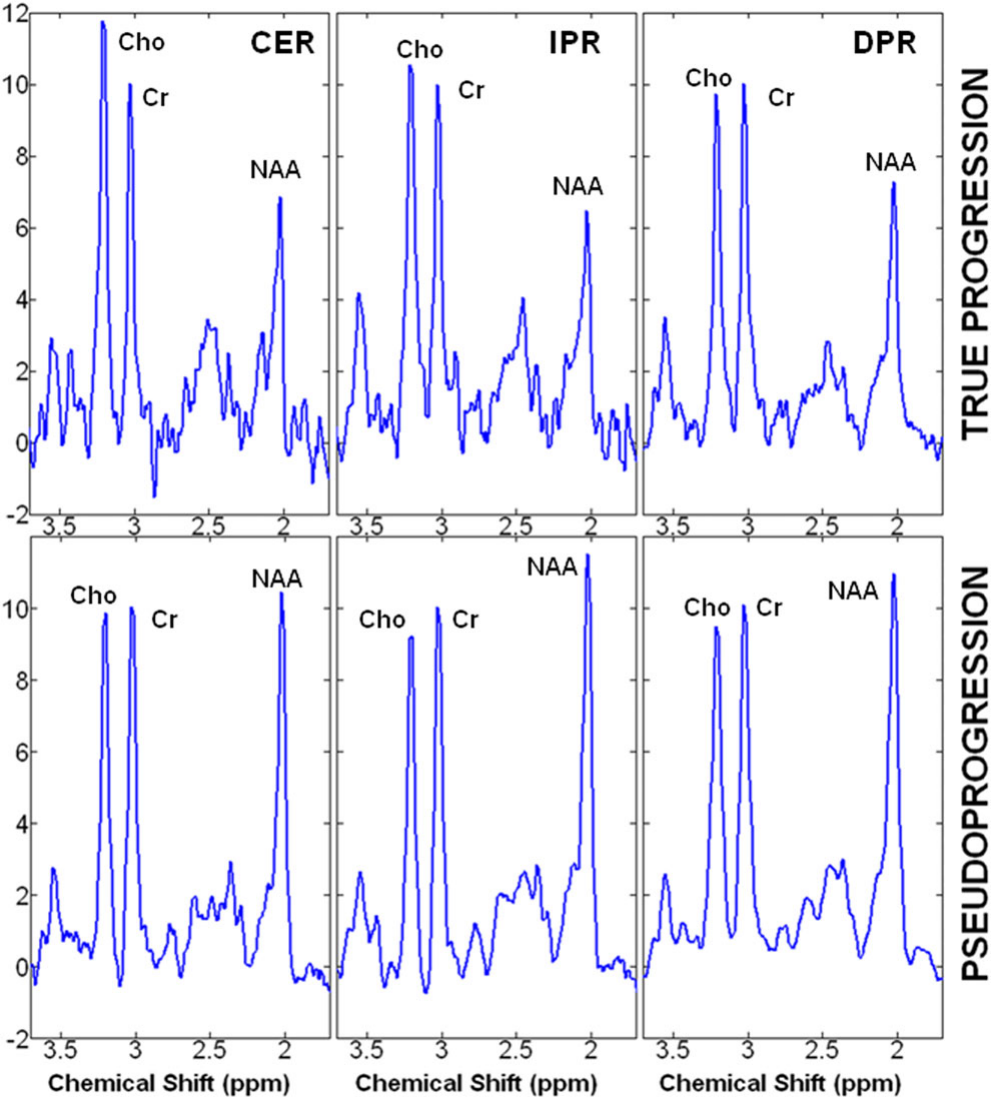
Summed ^1^H MRS spectra encompassing the entire volumes of different regions of neoplasms (CER, IPR, and DPR) from the TP and PsP patients shown in Figure 1. The number of voxels for these summed spectra from TP and PsP patients was CER 55 versus 41, IPR 75 versus 46, and DPR 75 versus 128, which explains the apparent differences in the signal to noise of these spectra. Significantly higher Cho/NAA and Cho/Cr from different regions of neoplasms were observed in TP patients compared with those with PsP

Representative anatomical images, Cho/NAA maps and corresponding re‐resected histologic photomicrographs from a TP and a PsP patient are shown in Figure 1. Summed ^1^H MRS spectra encompassing the entire volumes of different regions of neoplasms (CER, IPR, and DPR) from the two patients shown in Figure 1 are presented in Figure 2.

**Figure 3:**
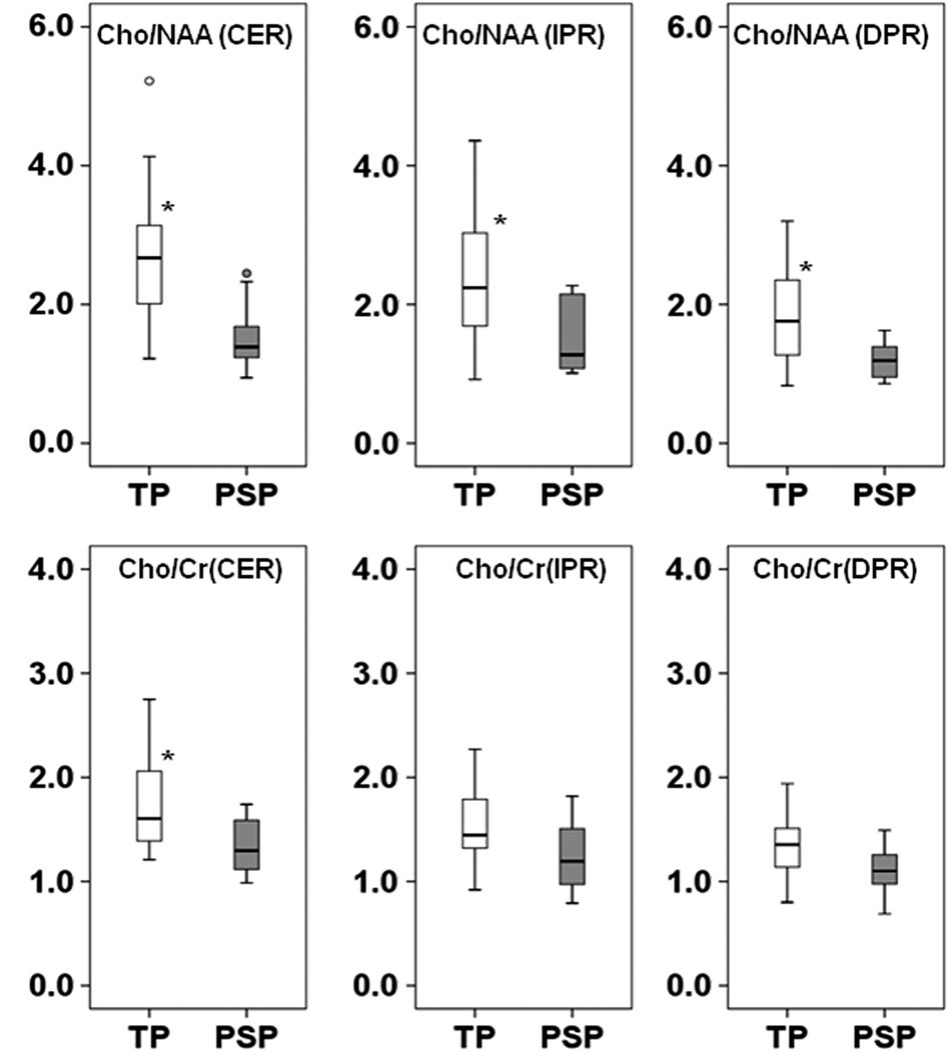
Box‐and‐whisker plots demonstrating the distribution of Cho/NAA and Cho/Cr from the different segmented regions, distinguishing TP from PsP patients at baseline. The bottom and top edges of boxes represent the 25th percentile and the 75th percentile values. The bands within the boxes represent 50th percentile (median) values. Whiskers display the range of data distribution. Outliers are marked with open circles (values 1.5 × box length from the 75th/25th percentiles). An asterisk indicates significant difference (*p* < 0.05) between the two groups

The distributions of Cho/NAA and Cho/Cr values from different regions of TP and PsP lesions from all patients are shown as box‐and‐whisker plots (Figure 3). Significantly higher Cho/NAA was observed from CER (2.69 ± 1.00 versus 1.56 ± 0.51, *p* = 0.003), IPR (2.31 ± 0.92 versus 1.53 ± 0.56, *p* = 0.030), and DPR (1.80 ± 0.68 versus 1.19 ± 0.28, *p* = 0.035) regions in TP patients compared with PsP. Similarly, significantly elevated Cho/Cr levels (1.74 ± 0.44 versus 1.34 ± 0.26, *p* = 0.023) from CER were observed in TP in comparison with PsP. Although higher Cho/Cr was also observed from IPR (1.54 ± 0.38 versus 1.24 ± 0.36, *p* = 0.062) and from DPR (1.35 ± 0.29 versus 1.10 ± 0.25, *p* = 0.054) in TP compared with PsP, these were not significant (*p* > 0.05).

**Figure 4:**
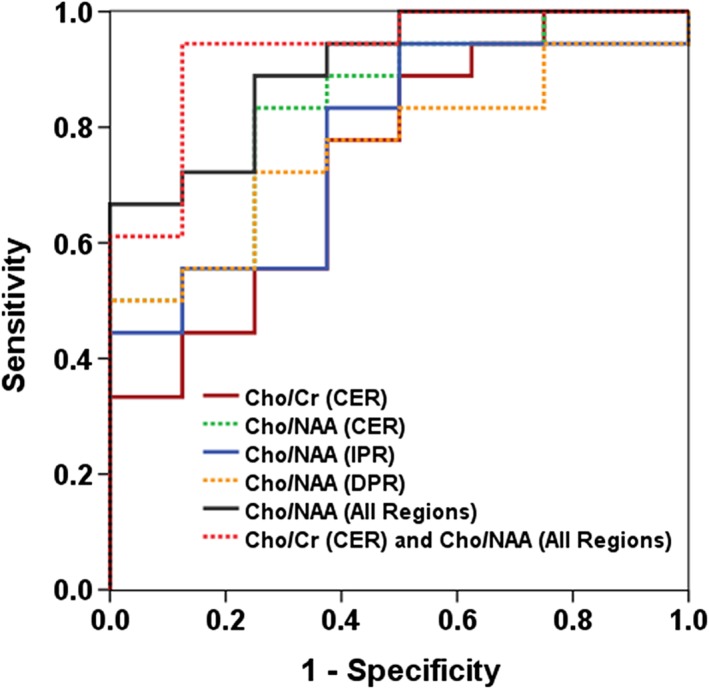
ROC curves for individual and combinations of different metabolite ratios. Using a univariate logistic regression model, Cho/NAA from CER provided the best AUC of 0.87 in discriminating TP from PsP patients with a sensitivity of 86% and specificity of 82%. After incorporation of Cho/Cr from CER and Cho/NAA from all regions (CER, IPR, and DPR) in multivariate logistic regression analyses the best model to distinguish the two groups of patients was observed, with an AUC of 0.93, a sensitivity of 94%, and a specificity of 86%. A leave‐one‐out cross‐validation test revealed that 92% of patients were correctly classified

ROC analysis revealed that Cho/NAA from CER was the best predictive parameter to distinguish TP from PsP, with a moderate accuracy (AUC = 0.83, Figure 4), a moderate sensitivity of 83%, and specificity of 75%. However, the best classification model for distinguishing TP from PsP was observed when Cho/Cr from CER and Cho/NAA from CER, IPR, and DPR were incorporated in the multivariate regression analyses as follows:

$$f\left(M1,M2\right)=\frac{1}{1+\exp\left(-\left(\beta 0+\beta 1M1\mathrm{CER}+\beta 2M2\mathrm{CER}+\beta 3M2\mathrm{IPR}++\beta 4M2\mathrm{DPR}\right)\right)}$$

where *M*_1_ = Cho/Cr, *M*_2_ = Cho/NAA, *β*_0_ = 11.22, *β*_1_ = −3.30, *β*_2_ = −6.86, *β*_3_ = 7.39, and *β*_4_ = −5.02. A substantially higher discriminant accuracy (AUC = 0.93, Figure 4), with a sensitivity of 94% and specificity of 87%, was observed in differentiating TP from PsP using the multivariate regression analysis. A summary of sensitivity and specificity for different metabolite ratios in distinguishing TP from PsP is presented in Table 1.

**Table 1:** AUC and associated sensitivity and specificity of normalized metabolite ratios using logistic regression analyses

Metabolite ratio (region)	AUC	Threshold value	Sensitivity (%)	Specificity (%)
Cho/Cr (CER)	0.74	1.38	78	63
Cho/Cr (IPR)	0.73	1.27	83	62
Cho/Cr (DPR)	0.74	1.18	72	75
Cho/NAA (CER)	0.83	1.73	83	75
Cho/NAA (IPR)	0.77	1.43	83	63
Cho/NAA (DPR)	0.76	1.30	72	75
Cho/NAA from all regions	0.90	0.18^a^	67	100
Cho/Cr (CER) and Cho/NAA from all regions	0.93	0.40^a^	94	87

a Threshold value of interaction factor from logistic regression model.

A leave‐one‐out cross‐validation test revealed that 92.3% of patients were correctly classified as TP or PsP using the multivariate logistic regression model.

When we compared metabolite ratios from different regions between TP and PsP at the follow‐up time point, significantly higher Cho/NAA from IPR (2.03 ± 0.50 versus 1.23 ± 0.28, *p* = 0.026), and a trend towards significance for Cho/NAA from CER (2.34 ± 0.91 versus 1.24 ± 0.50, *p* = 0.056), were observed in TP patients compared with those with PsP. Additionally, significantly higher Cho/Cr from CER (1.91 ± 0.33 versus 1.26 ± 0.14, *p* = 0.025) and IPR (1.80 ± 0.19 versus 1.20 ± 0.11, *p* = 0.036) were observed in TP than in PsP patients. While TP patients exhibited a median increase of 13.6% ± 59.1% in Cho/NAA from CER, PsP patients demonstrated a decline with a median value of −17.2% ± 44.7% at the follow‐up time point. Similarly, a median increase of 9.21% ± 55.7% in Cho/NAA from IPR in TP patients but a decrease of −18.6% ± 40.1% in PsP patients was observed.

## 4 DISCUSSION

This study demonstrates the utility of high spatial resolution 3D‐EPSI in distinguishing TP from PsP with a discriminatory accuracy of over 90%; this has a potential for improving management of GBM patients over routine clinical practice, which relies on RANO criteria. The differentiation between TP and PsP is critical, since TP patients are generally considered for repeat surgery, if feasible, or they may be offered alternative treatment strategies including enrollment in clinical trials involving novel treatment strategies such as TTFields^30^ or immunotherapy.^31^ On the other hand, PsP patients are usually continued on adjuvant TMZ therapy with standard follow‐up MRI at regular intervals. Accurate diagnosis may benefit proper enrollment of patients for clinical trials, which are often limited to recurrent tumors.

Classical radiation necrosis represents a variant of post‐treatment effects that differs from PsP in the time of onset and degree of severity.^17^ Although the underlying pathophysiological mechanism of PsP remains poorly understood, some studies indicate that PsP and radiation necrosis share similar histopathological and molecular features.^16^ Using ^1^H MRS, some studies^13^, ^14^, ^15^ have demonstrated that increases in Cho/NAA and/or Cho/Cr ratios are significantly higher in patients with recurrent tumors than those with radiation necrosis (RN). Since the pathogeneses of PsP and RN are similar,^16^ it may be speculated that these two entities would exhibit similar metabolite patterns, such that widely published reports^13^, ^14^, ^15^ of abnormal metabolite pattern from RN may be extrapolated to understand differences between TP and PsP. In agreement with our findings, the authors of a previous case series study^18^ also observed lower Cho/NAA ratio in PsP than TP.

Most of the previous studies^13^, ^14^, ^15^, ^18^ employed single voxel or single slice multi‐voxel ^1^H MRS methods to monitor post‐treatment changes in GBM, and were hence constrained by the limited spatial coverage of the neoplastic lesion. While analyzing single or multivoxel ^1^H MRS data from CER of a neoplasm, the sampled voxels might include the edges of the necrotic regions or peritumoral regions or both. Thus, metabolite levels in such lesions might be influenced by contributions from different tissue compartments of a neoplasm. By contrast, the 3D‐EPSI method used in the present study provides metabolite maps with better spatial resolution in comparison with single voxel or multivoxel ^1^H MRS sequences that can be spatially co‐registered to anatomical images to facilitate mapping of metabolite alterations from different regions of a neoplasm with less probability of partial volume averaging,^19^ thus projecting a more comprehensive representation of a tumor's true spatial extent. It is interesting to note that, despite similar tumor volumes (measured routinely using FLAIR abnormality), the Cho/NAA 3D volumes clearly showed a difference between TP and PsP patients (data not shown). This volumetric information may have significant impact on management, as it can provide more accurate tumor margins for the surgeon, assist in radiation planning, or be individually tailored for planning location‐sensitive treatment modalities such as TTFields.^26^

We observed significantly higher Cho/NAA and Cho/Cr from the CER regions in TP, which may be attributed to either an increase in Cho content (suggesting increased membrane turnover secondary to cell proliferation), a decrease in NAA (suggesting neuronal destruction and/or displacement) and Cr (suggesting altered energy metabolism), or a combination of these events.^32^ ROC analyses demonstrated that Cho/NAA from CER was the best independent predictor (AUC = 0.83, sensitivity = 83%, and specificity = 75%) in identifying TP or PsP. Higher Cho/NAA from peritumoral regions (IPR and DPR) in TP compared with PsP patients suggests that TP lesions were associated with a greater degree of neoplastic infiltration and/or greater damage to neuronal integrity in the regions beyond the CER. These findings suggest that mapping of metabolite ratios from peritumoral regions should also be considered when distinguishing TP from PsP, which is only possible with a 3D spectroscopic imaging scheme. Assessment of the true extent of tumor spread would facilitate the formulation of more aggressive treatment regimens for the management of patients with TP.

Using a combined analysis of Cho/Cr and Cho/NAA from CER, a meta‐analysis^33^ reported wide range in the sensitivity (36%**–**90%) and specificity (55%**–**83%) in discriminating recurrent tumors from radiation necrosis. Our discriminatory analysis showed that incorporation of Cho/Cr from CER and Cho/NAA from the tumor and peritumoral regions provided a higher accuracy of 93% in distinguishing TP from PsP, with a sensitivity of 94% and specificity of 87%. These findings further support the notion that analysis of metabolite ratios from different regions of neoplasms using a volumetric EPSI approach provides better discriminatory power than an individual region in stratifying patients with TP from PsP.

The RANO Working Group^4^ has suggested that radiological response at the initial presentation of a contrast enhancing lesion should persist for at least 4 weeks on follow‐up imaging before it can be considered as true response (TP or PsP). Though a small number of patients were included for the follow‐up time point in the present study, observation of continuously increasing Cho, within a month, in TP patients suggests measurable proliferation of active neoplastic cells within the tumor bed. However, the differences in the percentage change in metabolite ratios at follow‐up relative to baseline were not significantly different between TP and PsP patients. Possible explanations include variability in treatment response in this relatively small sample. Serial studies with 3D‐EPSI with a greater number of patients would help in tracking longitudinal changes in metabolite ratios.

Several data analysis methods^34^, ^35^ have been proposed to represent ^1^H MRS data for studying brain tumor metabolism and treatment response. These include both normalized and non‐normalized ways of reporting metabolite ratios. Given that there were no significant differences in the Cho/Cr and Cho/NAA ratios from contralateral normal brain parenchyma between TP and PsP patients, we normalized the metabolite ratios from different regions of neoplasms with respect to similar metabolite ratios from contralateral normal brain parenchyma in the present study. We did not investigate the differences in metabolite ratios from ipsilateral regions of neoplasms between TP and PsP patients, as we believe that normalized metabolite ratios, as reported in the present study, account for the inter‐subject variability in metabolite levels and facilitates unbiased group comparison. Our supposition is corroborated by an earlier study,^36^ in which normalized metabolite ratios were reported to be better than non‐normalized metabolite ratios in characterizing brain tumors. In another study,^15^ normalized metabolite ratios provided improved discrimination of recurrent tumors from RN than non‐normalized metabolite ratios.

Diffusion and perfusion imaging based MRI methods have also been used to differentiate TP from PsP. Using perfusion imaging, some studies^37^, ^38^ have reported sensitivity of 62%–90.2% and specificity of 77.8%–91.1% in differentiating TP from PsP. These studies suggest that increased relative cerebral blood volume (rCBV) may not necessarily be associated with TP, and local inflammatory response, secondary to infiltration of lymphocytes and macrophages, may also result in elevated rCBV. Some studies^39^, ^40^ have also reported the utility of various diffusion imaging parameters in distinguishing TP from PsP, with mixed sensitivities (71%–88%) and specificities (75%–90%), probably due to heterogeneity of the tissue. Additionally, exploiting the combined strengths of perfusion and diffusion imaging, recent studies^41^, ^42^, ^43^ have documented substantial increase in specificity (95%–100%), albeit with moderate sensitivity (76%–82%), in differentiating TP from PsP. Taken together, these studies provide a notion that perfusion and diffusion imaging techniques may also be promising in discriminating TP from PsP. However, variable degrees of success associated with these techniques motivated us to investigate the potential of ^1^H MRS as an additional alternative imaging modality in differentiating TP from PsP, as one study^44^ suggested that ^1^H MRS is better than diffusion imaging in evaluation of treatment response in patients with gliomas. Future studies combining diffusion, perfusion, and 3D‐EPSI may be needed to evaluate whether these imaging methods provide synergistic and complementary information towards better diagnosis.

Though promising, the results of our initial experience should be treated with caution, as these findings are from a relatively small number of patients. Although we did not have any selection bias for recruiting patients in the present study, the ratio of TP to PsP patients was almost 2:1, which may introduce some unintentional bias. However, it should be noted that the relative proportion of TP and PsP patients in our study is in line with the reported incidence rates of PsP in all GBM patients undergoing CCRT.^2^

In conclusion, 3D‐EPSI is a potentially valuable and accurate non‐invasive tool for differentiating TP from PsP. These results may have important clinical implications for guiding management decisions in the course of treatment for GBM patients. However, our findings should be validated in a blinded prospective study using threshold metabolite ratios from different regions of neoplasms as obtained in the current study in distinguishing TP from PsP.

## FUNDING INFORMATION

This work was supported by National Institutes of Health Grant 1R21CA170284 (HP) and R01EB016064 (AAM).
